# A New-Onset Facial Lesion in a Hospitalized COVID-19 Patient

**DOI:** 10.7759/cureus.38442

**Published:** 2023-05-02

**Authors:** Emelie E Nelson, Morgan A Rousseau, Cassandra A Mohr, Rashid M Rashid

**Affiliations:** 1 Dermatology, John P. and Kathrine G. McGovern Medical School at UTHealth, Houston, USA; 2 Dermatology, Mosaic Dermatology, Houston, USA

**Keywords:** pyoderma gangrenosum, prevention, skin, facial, pressure ulcer, covid-19

## Abstract

Pressure ulcers form when skin is compressed against a bony prominence, often in the context of prolonged supine or prone-based care. Hospitalized, bedridden patients are at the highest risk of this complication, especially when preventative measures like regular rotational bed treatment are not employed. In this case report, we present a rare case of a COVID-19-related facial pressure ulcer that occurred in the context of regular rotational bed treatment. The lesion was managed by wound care and allowed to heal by secondary intention. Ultimately, we hope that this manuscript will raise awareness for this atypical ulcer location, especially as prone-position treatment approaches take hold.

## Introduction

Pressure ulcers are characterized by the breakdown of skin and underlying tissue from prolonged compression of the skin against a bony prominence [1]. Hospitalized, bedridden patients are at the highest risk of this complication on the posterior body due to prolonged supine-based care [1]. In this case report, we present a rare case of a COVID-19-related facial pressure ulcer that occurred in the context of regular rotational bed treatment. Our goal is to increase awareness of this atypical ulcer location, especially as we see more prone-position treatment approaches take hold.

## Case presentation

**Figure 1:**
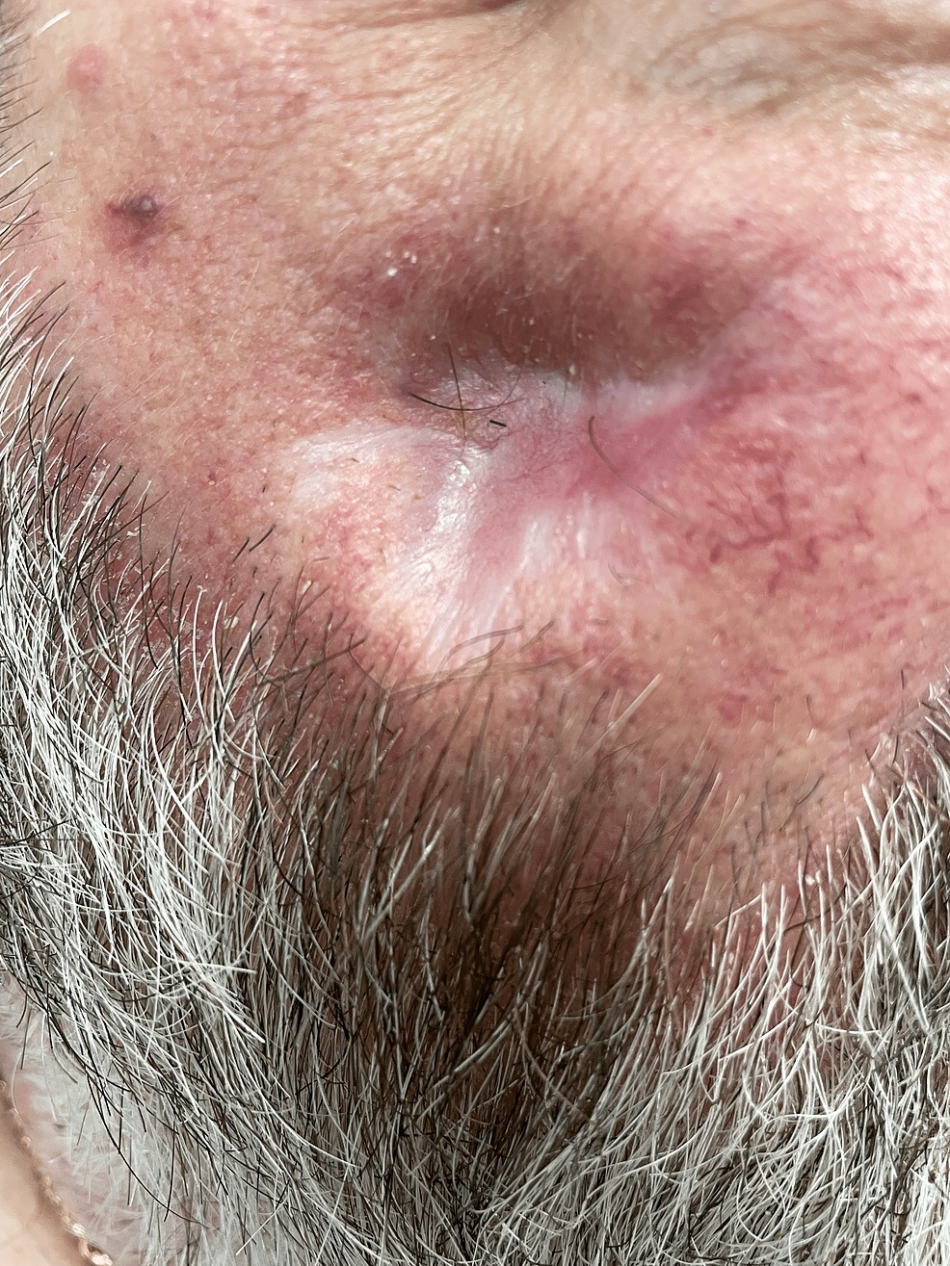
The patient's right mandible and associated healing pressure ulcer on follow-up presentation to dermatology.

A 62-year-old male with a past medical history significant for diabetes and hypothyroidism presented to dermatology for follow-up of a COVID-19-related pressure ulcer. The patient had recently been admitted to the ICU for six weeks with a COVID-19 infection, during which time he received regular rotational bed treatment. However, on week three, a non-blanchable, erythematous lesion with intact skin was noted along the patient’s right mandible, consistent with a stage 1 facial pressure ulcer. No purulence was observed at that time; however, the patient reported significant pain in the affected area. The lesion was managed by wound care, with regular dressing changes, and allowed to heal by secondary intention. Physical examination on follow-up evaluation revealed a well-healing ulcer with the presence of scarring (Figure 1). The patient denied residual pain, numbness, or any other associated symptoms.

## Discussion

The clinical presentation of pressure ulcers varies among anatomic regions and ulcer stages but typically begins as an erythematous patch (stage 1) before progressing to a red, swollen plaque with or without blisters (stage 2). It then ulcerates (stage 3) and deepens, exposing fat, muscle, and bone with or without the presence of an eschar (stage 4) [2]. Intrinsic risk factors for the development of pressure ulcers include immobility, diabetes mellitus, obesity, and hypertension. Extrinsic factors, such as lying on hard surfaces and poorly fitting prostheses, further promote ulcer formation [1,2]. The majority of cases occur in patients over the age of 70, with extended hospital stays (more than five days) and hospitalizations requiring ICU admission [2].

While facial pressure ulcers are rare, they have been reported in ICU patients hospitalized with COVID-19 [3,4]. Patients with COVID-19 complicated by acute respiratory distress syndrome are placed in a prone position, which can lead to persistent facial pressure, especially at the level of bony structures [3]. It has been hypothesized that prolonged prone positioning, coupled with hypoxemia, microvascular injury, and thrombosis, greatly increases the risk of facial pressure ulcer development in this patient population.

Before the diagnosis of our patient’s facial pressure ulcer, ulcers of other etiologies and morphologic mimics were considered, including neurogenic ulcers, venous stasis ulcers, ischemic ulcers, and pyoderma gangrenosum. Though our patient had risk factors for the development of neurogenic ulcers due to his diabetes, they typically present on the bottom of the foot [5]. Stasis ulcers, which have a predilection for the medial malleolus, and ischemic ulcers, which predominantly affect the feet, were also ruled out due to location [5]. Finally, pyoderma gangrenosum is a diagnosis of exclusion and cannot be confirmed until other possible causes of ulceration have been ruled out.

When positioning a patient in a prone position due to ARDS, the use of thin, soft silicone foam dressings has been proposed as a possible method of preventing facial pressure ulcers [6]. Regular changes in head position, as was implemented for our patient, and changing the position of the breathing tube are also important preventative strategies [3]. If a facial pressure ulcer develops, pressure on the area of the wound needs to be relieved promptly. The necrotic tissue should then undergo proper debridement, allowing the wound to heal by secondary intention with a paraffin gauze dressing or alginate, depending on the extent of the injury. 

## Conclusions

This case report presents one of the few cases of COVID-19-related facial pressure ulcers described in dermatology literature, especially in the context of rotational bed treatment. It is important that physicians are aware of this potential complication of prone positioning and that preventative measures, such as regular changes to head position and tube placement, as well as the use of soft silicone foam dressings, are taken to minimize the risk of facial pressure formation. However, if a facial pressure ulcer does form, pressure should be removed from the affected area, and appropriate interventions should be taken promptly, as early intervention can greatly reduce patient morbidity.
